# Chinese herbal medicine (Guben Qushi Huayu formula) combined with Ixekizumab in reducing psoriasis vulgaris relapse: Protocol for a mixed-methods research study

**DOI:** 10.3389/fphar.2025.1551001

**Published:** 2025-03-21

**Authors:** Ziqing Li, Kewen Guan, Hao Deng, Shuyan Ye, Jingwen Deng, Danni Yao, Yuhong Yan, Haiming Chen, Chuanjian Lu, Jingjie Yu

**Affiliations:** ^1^ The Second Clinical Medical College of Guangzhou University of Chinese Medicine, Guangzhou, China; ^2^ The Second Affiliated Hospital of Guangzhou University of Chinese Medicine, Guangdong Provincial Hospital of Chinese Medicine and Guangdong Provincial Academy of Chinese Medical Sciences, Guangzhou, China; ^3^ State Key Laboratory of Dampness Syndrome of Chinese Medicine, Guangdong-Hong Kong-Macau Joint Lab on Chinese Medicine and Immune Disease Research and Guangdong Provincial Key Laboratory of Clinical Research on Chinese Medicine Syndrome, Guangzhou, China

**Keywords:** psoriasis vulgaris, relapse, Ixekizumab, Guben Qushi Huayu formula, mixed-method research, protocol

## Abstract

**Introduction:**

Psoriasis vulgaris (PV) is an inflammatory, chronically relapsing dermatological disease associated with significant comorbidities. Ixekizumab is recommended as the first-line therapy for severe PV, but encounters persistent challenges with relapse after treatment discontinuation. In clinical practice, Chinese herbal medicine (CHM) including Guben Qushi Huayu formula (GQHF) has been demonstrated effective in reducing PV relapse. However, there remains a scarcity of high-level evidence-based study in this respect. Therefore, this study aims to preliminarily evaluate the feasibility and acceptability of Ixekizumab combined with GQHF in reducing PV relapse.

**Methods and analysis:**

This study employs a mixed-method research (MMR) design, encompassing both quantitative and qualitative studies. The quantitative study consists of a randomized controlled trial involving 50 participants with severe PV, who will be randomly allocated to the intervention group (Ixekizumab plus GQHF) and the control group (Ixekizumab plus GQHF placebo) in a 1:1 ratio. Relapse rate is the primary endpoint. The qualitative study involves semi-structured interviews to concurrently explore the acceptability of the application of Ixekizumab combined with GQHF among the enrolled participants.

**Discussion:**

This pilot study utilizes MMR to investigate the effect of Ixekizumab combined with GQHF in reducing PV relapse. The findings are expected to provide valuable clinical evidence and a novel therapeutic option for PV. Moreover, it is our intention to conduct a larger MMR trial to further strengthen the clinical evidence and broaden the application of Ixekizumab in combination with GQHF.

**Clinical Trial Registration:**

https://www.chictr.org.cn/index.html, identifier ChiCTR2100054950.

## 1 Introduction

Psoriasis vulgaris (PV) is a chronic immune-mediated relapsing inflammatory dermatological disease (Armstrong and Read, 2020), with an increasing prevalence of approximately 2.0%–4.0% of the worldwide population (Parisi et al., 2020; Armstrong et al., 2021; Mou et al., 2022). Given the aggravation and relapse of PV, it gives rise to multiple comorbidities such as psoriatic arthritis, cardiovascular and cerebrovascular disorders, malignant tumors (Elmets et al., 2019; Griffiths et al., 2021), which led to a substantial economic burden on patients and society (Brezinski et al., 2015; Hamilton et al., 2015). Alarmingly, during the prolonged period of uncontrolled disease, patients with PV are susceptible to psychological disorders such as depression and suicidal tendencies (Egeberg et al., 2019; Bardazzi et al., 2022; Poot, 2017).

Currently, the underlying mechanisms of PV remain incompletely understood. The interleukin-23 and T cell type 17 (IL-23/Th17) pathway is recognized as a potential therapeutic target for PV (Ghoreschi et al., 2021; Sharma et al., 2022), with downstream cytokines such as IL-17 and TNF-ɑ being pivotal in the treatment of PV (Bachelez, 2015; Wcislo-Dziadecka et al., 2016). With the increasing occurrence of severe PV,standard biomedical treatments such as IL-17A inhibitors, TNF-ɑ inhibitors and IL-12/23 inhibitors have been extensively utilized for PV (Dapavo et al., 2022; Bai et al., 2019; Mahil and Smith, 2019). Nowadays, Ixekizumab, as a kind of IL-17A inhibitor, has been applied for severe PV in clinical practice (Tsuda et al., 2016; Craig and Warren, 2020; Gordon et al., 2016). Owing to the rapid onset and notable efficacy, international guideline for PV (Nast et al., 2021) indicates that Ixekizumab is a recommended therapeutic regimen for severe PV. Additionally, several published head-to-head randomized controlled trials have reported that, when compared with IL-12/23 inhibitors and TNF-ɑ inhibitors such as Guselkumab, Ustekinumab and Adalimumab, Ixekizumab showed superior in rapidly relieving psoriatic lesion after 12-week treatment (Blauvelt et al., 2020; Reich et al., 2017; Mease et al., 2020). However, during the treatment discontinuation after relieving psoriatic lesions, Ixekizumab faces challenge of disease relapse, which is the common issue to other biological therapies (Masson et al., 2022; Wang et al., 2020). To date, no clinical consensus and guidelines on reducing and administering PV relapse has been published, which is a gap in the management of severe PV.

Chinese herbal medicine (CHM) has been recommended as an alternative therapy in guidelines (Elmets et al., 2021; Committee on Psoriasis, 2023) and has been utilized for the treatment of PV for centuries (Yang et al., 2023). Relevant studies have indicated that CHM can alleviate psoriatic symptoms and decrease the relapse rate (Chen et al., 2020; Lu et al., 2023; Li et al., 2023; Wang et al., 2024; Huang et al., 2024). Furthermore, when combined with standard biomedical treatment for PV, such as narrow-band UVB, methotrexate, acitretin and cyclosporin, CHM has exhibited superiority in reducing PV relapse (Li et al., 2019; Zhang et al., 2009; Wang et al., 2024). Guben Qushi Huayu Formula (GQHF) is a prescribed CHM that is widely applied for the treatment of PV in clinical practice. Network pharmacology and metabolomics have elucidated the potential action mechanism of GQHF (Xiang, C, 2021). Our team has optimized the efficacy and safety profile of GQHF based on previous clinical studies (Li et al., 2024; Lu, Y et al., 2021; Yu, J et al., 2021). Experimental studies have also reported the therapeutic mechanisms of GQHF in the treatment of PV (Chen, H et al., 2024; Xiang, C, 2021; Chen, W, 2022). Specific to the patients with severe PV, our team has previously conducted two single-arm trials investigating the effect of GQHF combined with IL-17A inhibitors, specifically Ixekizumab and Secukinumab. Within these trials, Ixekizumab and Secukinumab were utilized separately in conjunction with GQHF. The findings indicated that the median time to disease relapse was prolonged when undergoing treatment with Ixekizumab and GQHF, compared to the regimen with Secukizumab and GQHF. Additionally, lower incidence of adverse events was observed in the treatment of Ixekizumab and GQHF.

Aligned with the therapeutic goals for PV (Amatore et al., 2019; Nast et al., 2021; Nast et al., 2020), reducing relapse after remission is a critical objective. Considering the rapid symptomatic relief provided by Ixekizumab and the relapse-reducing properties of GQHF, we aim to conduct a mixed-method research (MMR) study to assess the effect of combining GQHF with Ixekizumab in reducing relapse of PV. We will carry out this study in accordance with the guidelines outlined in the Standard Protocol Project Statement: Recommendations for Interventional Trials (SPIRIT) (Butcher et al., 2022). The findings from this study are expected to provide evidence supporting the combined use of GQHF and Ixekizumab in reducing PV relapse.

## 2 Methods and analysis

### 2.1 Study objective and design

**FIGURE 1:**
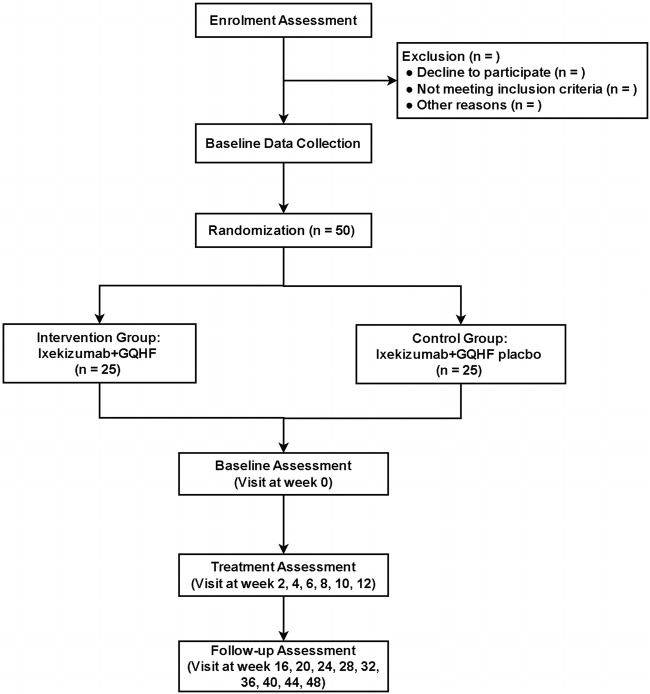
The CONSORT flow diagram of randomized controlled trial. GOHF, Guben Qushi Huayu Formula.

**FIGURE 2:**
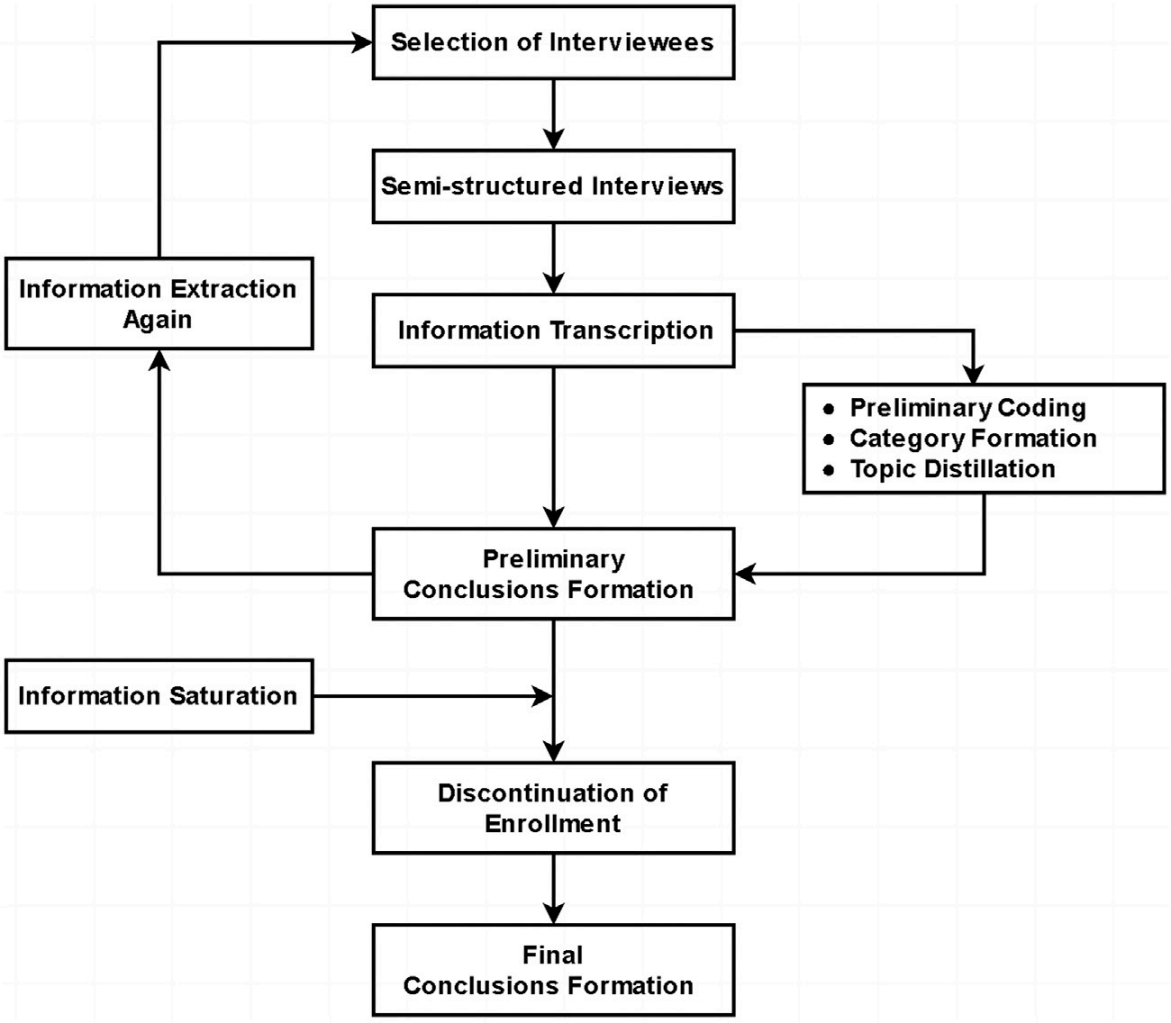
Flow diagram of study of semi-structured interview.

This MMR study aims to preliminarily evaluate the effect of GQHF combined with Ixekizumab in reducing relapse of PV. The MMR study design includes both a quantitative study and a qualitative study. The quantitative study constitutes a randomized, double-blind, placebo-controlled trial to evaluate the feasibility of combined therapy of GQHF and Ixekizumab (Figure 1). The qualitative study is semi-structured individual interviews to explore the acceptability of the combined therapy among the enrolled participants (Figure 2).

### 2.2 Quantitative study (A randomized controlled trial)

#### 2.2.1 Study setting

The randomized controlled trial will be conducted at Guangdong Provincial Hospital of Chinese Medicine in Guangzhou, China. All participants will be recruited from public or dermatology outpatient departments.

#### 2.2.2 Inclusion criteria

(1) Patients aged 18–70, with no restriction to gender.
(2) Patients diagnosed with severe PV, with psoriasis area severity index (PASI) ≥ 10 or body surface area (BSA) ≥ 10%.
(3) Patients with indication of Ixekizumab and without history of inflammatory bowel disease.
(4) Patients who have signed the informed consent.

#### 2.2.3 Exclusion criteria

(1) Pregnant or lactating women or those planning pregnancy during the study.
(2) Patients with self-rating anxiety scale score >50 or self-rating depression scale standard score >53, or other mental disorders.
(3) Patients with basic diseases (such as cardiopulmonary diseases) that cannot be controlled with conventional medication alone; patients with severe infection, (tuberculosis, hepatitis or other infectious history), lymphocyte proliferation, hematopoietic system abnormalities and tumor; patients with severe electrolyte imbalances; patients with immune deficiency and hypersensitivity. Additionally, the following clinical test indicators will result in exclusion: alanine aminotransferase (ALT) or aspartate aminotransferase (AST) levels that are three times the normal value; creatinine levels that are 1.5 times the normal value; a positive serological test for human immunodeficiency virus; any key blood routine indicators lower than the normal range; and patients deemed unsuitable by researchers due to other abnormal laboratory findings.
(4) Patients who are allergic to the medication or ingredients used in this study.
(5) Patients who have participated in other clinical trials within 4 weeks.
(6) Patients who have used CHM, Chinese herbal products or topical therapy (such as antibiotics, external corticosteroids, vitamin D3 analogues, calcineurin inhibitors) within 2 weeks; patients who have used conventional systemic therapies (methotrexate, ciclosporin A, acitretin, fumarates, et al.) or ultraviolet treatment within 4 weeks; patients who have received biomedical treatment within 5 half-life periods (17.5 days of Etanercept, 50 days of Infliximab, 70 days of Adalimumab, 105 days of Ustekinumab, 135 days of Secukinumab, 65 days of Ixekizumab, et al.).
(7) Patients who are considered unsuitable for this study by researchers.

#### 2.2.4 Withdrawal criteria

The initial assessment consists of screening based on inclusion and exclusion criteria, informed consent interpretation, and laboratory tests. Indicators for enrolment assessment include PASI, BSA, physicians global assessment (PGA), as well as pruritus scores on the visual analogue scale (VAS). Interpretation of informed consent is performed at the beginning, with explanations provided for any questions arising from participants. In addition, written information and verbal explanation of this study will be provided. Participants are free to withdraw from this study at any time, with or without reasons, and that will belong to the cases of dropout before completing the entire study.

#### 2.2.5 Sample size estimation

This is a pilot study aimed at preliminarily evaluating the effect of GQHF combined with Ixekizumab for PV on relapse reduction. Notably, there is a lack of relevant studies providing basic data for sample size calculation of this study. Additionally, published research (Sim and Lewis, 2012) recommends enrolling at least 50 participants in a dual-arm pilot randomized controlled trial when there is high confidence. Therefore, this study aims to enroll 50 participants for preliminary assessment.

#### 2.2.6 Randomization and blinding

Recruited participants will be randomly allocated to the intervention group and the control group in a 1:1 ratio. Computer-generated random list and permuted block size for center-stratified method are generated with SAS 9.2 software. Participants and researchers will be rigorously blinded to the group allocation. According to the blinding requirement of study, the granule of GQHF and GQHF placebo will be packaged and labeled identically with randomized codes by an independent researcher. The blinding codes are generated after randomization. Blinding will be maintained until data validation and editing are complete, or in the onset of serious adverse events.

#### 2.2.7 Intervention

Ixekizumab, a kind of humanized monoclonal biomedical treatment targeting IL-17A, has been approved for PV by FDA in the United States (Menter et al., 2019) and in China (Chinese Society of Dermatology, 2021). In addition, Ixekizumab is marketed and frequently utilized in clinical practice. Guben Qushi Huayu Formula (GQHF), a kind of CHM, has demonstrated effective for PV in previous clinical and experimental studies (Chen et al., 2024; Zhang et al., 2024; Chen, 2022; Xiang, 2021). The components of GQHF are presented in Table 1.

**TABLE 1:** Component of Guben Qushi Huayu formula.

Scientific name	Pharmaceutical name	Produced from	Doses (g)
*Astragalus membranaceus* (Fisch.) Bge. [Fabaceae]	*Astragali Radix*	Dry rhizoma	30
*Rehmannia glutinosa Libosch*. [Scrophulariaceae]	*Rehmanniae Radix Praeparata*	Dry rhizoma	15
*Atractylodes macrocephala* Koidz. [Asteraceae]	*Atractylodis Macrocephalae Rhizoma*	Dry rhizoma	15
*Smilax glabra* Roxb. [Liliaceae]	*Smilacis Glabrae Rhizoma*	Dry rhizoma	30
*Paeonia lactiflora* Pall. [Ranunculaceae]	*Paeoniae Radix Rubra*	Dry rhizoma	15
*Curcuma phaeocaulis* Val. [Zingiberaceae]	*Curcumae Rhizoma*	Dry rhizoma	10
*Trionyx sinensis* Wiegmann [Trionychidae]	*Trionycis Carapax*	Tergum	30
*Prunus mume* (Sieb.) Sieb. et Zucc. [Rosaceae]	*Mume Fructus*	Fruit	15
*Sarcandra glabra* (Thunb.) Nakai [Chloranthaceae]	*Sarcandrae Planta*	Herbal	15

The intervention group will be administered GQHF granule (Jiangyin, Jiangsu Province, China) after meals twice daily for 12 weeks. Simultaneously, Ixekizumab (Taltz) will be subcutaneously injected with initial dose of 160 mg at week 0 followed by 80 mg fortnightly until week 12. A 36-week follow-up will be carried out subsequently to further observe the clinical effect.

The control group will receive GQHF placebo granule (Jiangyin, Jiangsu Province, China) twice daily for 12 weeks, which is identical with GQHF in appearance and taste. Meanwhile, injection of Ixekizumab (Taltz) will be administered according to the same usage and dosage with the treatment group. The 36-week follow-up period will continue later.

#### 2.2.8 Outcomes

##### 2.2.8.1 Primary outcome

Rate of relapse is the primary outcome, wherein relapse is defined as the loss of response of at least 75% improvement in PASI score from baseline (PASI-75) during the follow-up period by participants who achieve PASI-75 after treatment (Warren et al., 2021; Mrowietz et al., 2015).

##### 2.2.8.2 Secondary outcomes

(1) Time to relapse, defined as the interval between the completion of the last treatment and the occurrence of disease relapse.
(2) Improvement in PASI score from baseline.
(3) Proportion of participants achieving PASI-75.
(4) Proportion of participants achieving at least 90% improvement in PASI score from baseline (PASI-90).
(5) Changes in PGA score.
(6) Changes in VAS score.
(7) Changes in Dermatology Life Quality Index (DLQI) score.
(8) Changes in Skindex16 score.

PASI-75, PASI-90, BSA and PGA are the recommended outcome measures for assessing the efficacy of PV (Elmets et al., 2021; Committee on Psoriasis, 2023). Regarding the assessment of quality of life, the DLQI is a validated and recognized measure involving aspects of symptoms, feelings, daily activities, recreation, work, study, personal relationships and therapy (Atwan et al., 2017; Mermin et al., 2016). Additionally, the Skindex 16 is another assessment scale that has been proven sensitive and highly applicable in the treatment of PV (Hopkins et al., 2023; He et al., 2014).

##### 2.2.8.3 Safety evaluation

All adverse events occurring during the study will be recorded, and any serious adverse event will be promptly reported to the Human Research Ethics Committee of Guangdong Provincial Hospital of Chinese Medicine, Guangzhou, China. Laboratory examinations will be conducted periodically, including routine blood tests, liver and kidney function tests, stool test, urine tests, chest radiography and 12-lead electrocardiography.

#### 2.2.9 Timeline of visits

**FIGURE 3:**
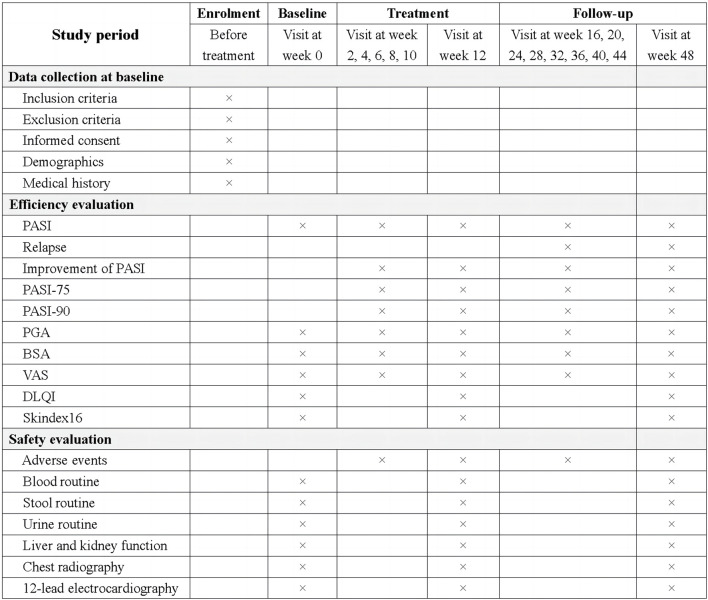
The schedule of study procedure. PASI, Psoriasis area and severity index; PASI-75, at 75% improvement in PASI score from baseline; PASI-90, at 90% improvement in PASI score from baseline; PGA, Physicians global assessment; BSA, Body surface area; VAS, Pruritus scores on the visual analog scale score; DLQI, Dermatology life quality index.

The assessment time points included four periods: enrolment, baseline, treatment, and follow-up. The study procedure is illustrated in Figure 3.

#### 2.2.10 Data collection and management

Data collection is conducted at each visit, covering the periods of enrolment, baseline, treatment and follow-up. Training on data recording will be provided to all research personnel prior to the commencement of the trial. Data from original documents, such as Case Report Form, will be stored in a password-protected dataset. A double-check will be conducted to ensure the accuracy of recorded data. Any correction to the written text will be documented and dated within the original file. Quality control of the finial data will be held in accordance with relevant national and industry standards.

#### 2.2.11 Statistical analysis

Statistical analyses will be conducted utilizing PASW Statistics 26.0 (IBM SPSS Inc, Armonk, New York, USA) and R statistical software version 4.4.1 by an independent statistician blinded to group allocation. Missing data will be involved with the last observation carried forward (LOCF) method. The primary analysis will be focus on the intention-to-treat (ITT) population, including all randomized subjects. Concerning efficacy analysis, frequency counts and percentages of participants in each arm will be calculated for categorical data (such as relapse rate), and group comparisons will be conducted using chi-squared test or Fishers exact test appropriately. Continuous variables will be presented as means with standard deviations for normally distributed data, or medians with interquartile ranges for non-normally distributed data. Group comparisons will be conducted by the t-test or Wilcoxon rank-sum test. For rank data (such as PGA score), intergroup comparison will be performed using the Wilcoxon rank sum test or Cochran-Mantel-Haenszel test. For repeated measures analysis, generalized estimating equations (GEE) or Cumulative Link Mixed Model (CLMM) will be employed to evaluate data across all follow-up time points. The Log-rank test will be utilized to compare the “time to relapse” and “time to achieve improvement” between two groups. All statistical tests will be conducted using a two-tailed hypothesis, with *p* values ≤0.05 considered indicative of statistical significance.

#### 2.2.12 Trial status

We planned to recruit participants from May 2022, with recruitment expected to be completed in January 2025. The follow-up period will finish in September 2025 and all clinical data will be locked in November 2025. This protocol was submitted before completion of recruitment.

### 2.3 Qualitative study (A semi-structured interview)

#### 2.3.1 Participants

Participants recruited in the randomized controlled trial will be enrolled in this semi-structured interview. Additional informed consent must be obtained prior to participation in this qualitative study.

#### 2.3.2 Data collection

The semi-structured interviews will be conducted individually either in a quiet waiting room or online, and will last between 30 and 60 min. During the interviews, participants will be encouraged to express their attitudes, expectations and experiences regarding the combined therapy of GQHF and Ixekizumab. Additionally, open-ended questions will be used to explore participants preferences and challenges related to this therapeutic regimen. Audio recording of each interview will be made to ensure the data authenticity.

#### 2.3.3 Date management

Transcriptions will be completed to convert the interview content into a written version using the language of the interviews. Any information that might reveal participants identities will be replaced with pseudonyms in the transcripts. Data saturation is achieved when no additional information is obtained from further interviews.

#### 2.3.4 Statistical analysis

Thematic content analysis will be conducted for data processing. Transcripts will be imported into QSR NVivo software for data management and coding. Researchers will apply “open coding” to each line of transcribed data to identify themes for this study. Preliminary analysis will be conducted immediately after each interview to determine if adjustments are needed to the interview guide. Researchers will meet at regular intervals to ensure the consistency in data analysis and to direct the next phase of analysis.

## 3 Discussion

The current method capable of rapidly and efficiently eliminating lesions of severe PV is standard biological treatment. Ixekizumab, recognized as an effective biomedical treatment targeting IL-17A, is internationally approved in the treatment of PV (Menter et al., 2019; Chinese Society of Dermatology, 2021) and is recommended as the first-line therapy for severe PV in published guideline (Nast et al., 2021). In addition to rapid lesion remission, Ixekizumab encounters significant challenges in disease relapse following treatment discontinuation. Published researches have demonstrated the median time to relapse for Ixekizumab is approximately 20.4 weeks (Masson et al., 2022; Wang et al., 2020). CHM has been widely applied for the treatment of PV in China, and GQHF as a form of CHM has been used in clinical practice for several decades. Our research team has conducted a variety of clinical and experimental studies to evaluate the efficacy and safety of GQHF (Li et al., 2024; Lu et al., 2021; Chen et al., 2024; Xiang, 2021).

To enhance the clinical application of GQHF for PV, further research is greatly needed, particularly regarding its single use and combined use. Patients with severe PV will be included in this study. This pilot study with MMR design aims to evaluate the effect of GQHF combined with Ixekizumab on relapse reduction in the treatment of PV. MMR, a study design that combines quantitative and qualitative approaches (Moorley and Cathala, 2019; Palinkas et al., 2011), provides comprehensive findings in various perspective, including objective assessment from physicians through quantitative method and individual subjective assessment from patients through qualitative research (Pineda-Canar et al., 2024; Wanqing et al., 2022). Consequently, a randomized, double-blind, placebo-controlled trial as a quantitative and semi-structured interviews as qualitative study will be carried out concurrently to evaluate the feasibility and acceptability of GQHF combined with Ixekizumab in reducing PV relapse.

However, there are also several limitations needed to be addressed. Firstly, patients older than 70 years old and younger than 18 years of age are excluded in this study, since it is unclear whether the combined treatment of GQHF and Ixekizumab is suitable for these age groups. Additionally, due to the absence of relevant published studies on the application of GQHF combined with Ixekizumab, the sample size calculation of this pilot study is based on the minimum sample size of dual-arm pilot trials (Sim and Lewis, 2012), which may affect the generalizability and reliability of the findings. Therefore, after completing this pilot study, we intend to conduct the next phase study with a larger sample size and multiple research centers as planned.

In summary, this pilot study aims to evaluate the feasibility and acceptability of combing GQHF with Ixekizumab to reduce PV relapse. To comprehensively investigate the effect of GQHF combined with Ixekizumab for PV, study design of MRR is utilized, containing a randomized controlled trial for feasibility assessment and a semi-structured interview for acceptability evaluation. The findings of this study may indicate a novel therapeutic option for PV in reducing relapse and provide preliminary data for further MMR study with larger sample and multiple centers.
